# Congenital muscular dystrophy: from muscle to brain

**DOI:** 10.1186/s13052-016-0289-9

**Published:** 2016-08-31

**Authors:** Raffaele Falsaperla, Andrea D. Praticò, Martino Ruggieri, Enrico Parano, Renata Rizzo, Giovanni Corsello, Giovanna Vitaliti, Piero Pavone

**Affiliations:** 1 Unit of Pediatrics and Pediatric Emergency, University Hospital “Policlinico-Vittorio Emanuele”, Catania, Italy; 2 Section of Pediatrics and Child Neuropsychiatry, Department of Clinical and Experimental Sciences, University of Catania, Catania, Italy; 3 Department of Biomedical and Biotechnological Sciences, University of Catania, Catania, Italy; 4 National Research Council—Section of Catania, Catania, Italy; 5 Department of Maternal and Child Health, University of Palermo, Palermo, Italy

**Keywords:** Muscular dystrophies, Congenital muscle diseases, Brain involvement, Muscle-eye-brain disease, Walker-Warburg syndrome, Fukuyama congenital muscular dystrophy

## Abstract

Congenital muscular dystrophies (CMDs) are a wide group of muscular disorders that manifest with very early onset of muscular weakness, sometime associated to severe brain involvement.

The histologic pattern of muscle anomalies is typical of dystrophic lesions but quite variable depending on the different stages and on the severity of the disorder.

Recent classification of CMDs have been reported most of which based on the combination of clinical, biochemical, molecular and genetic findings, but genotype/phenotype correlation are in constant progression due to more diffuse utilization of the molecular analysis.

In this article, the Authors report on CMDs belonging to the group of dystroglycanopathies and in particular on the most severe forms represented by the Fukuyama CMD, Muscle-Eye-Brain disease and Walker Walburg syndrome.

Clinical diagnosis of infantile hypotonia is particularly difficult considering the different etiologic factors causing the lesions, the difficulty in localizing the involved CNS area (central vs. peripheral) and the limited role of the diagnostic procedures at this early age.

The diagnostic evaluation is not easy mainly in differentiating the various types of CMDs, and represents a challenge for the neonatologists and pediatricians. Suggestions are reported on the way to reach a correct diagnosis with the appropriate use of the diagnostic means.

## Background

**Fig. 1:**
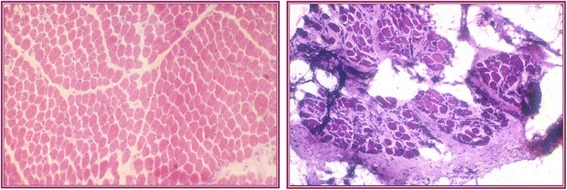
Archive’s photo. Histological features of the patient with WWS showing the typical dystrophic lesions at the beginning of the disease with initial lesions (*left*) and after 14 months with clear dystrophic features (*right*)

Muscular dystrophies (MDs) encompass a group of disorders presenting with skeletal muscle weakness, peculiar histological muscle alterations and consisting in abnormal muscular architecture with loss of muscular tissue (Fig. 1) and variable outcomes. In some cases, involvement is not limited to muscles, but it may affect other organs (e.g., heart). Until recently, MDs were clinically distinguished into six categories represented by Duchenne MD (DMD) and Becker MD; limb-girdle MD (LGMD); distal myopathies; congenital MD (CMD); facio-scapulo-humeral MD; and myotonic dystrophy. The recent diffusion of genetic investigations has notably enlarged the knowledge in the field of muscle disorders, and, at present, more than 50 forms of MDs are distinguished on the basis of specific genetic mutations, each one with a different pattern of muscle distribution, different involvement of body organs other than the skeletal muscles, and variable course. As recently demonstrated in most of the MDs, a specific genotype/phenotype correlation has been reported, even if in a minor number of cases examples of a same gene expressing multiple phenotypes have been reported [1].

Congenital Muscular Dystrophies (CMDs) are an uncommon group of MD disorders characterized by early onset of muscular weakness within 1 year of age and manifesting with more or less severe neonatal hypotonia as well as histologically presenting with features of dystrophic lesions [2]. Muscle biopsies in patients with CMDs show considerable variability but always exhibit the characteristic pattern of dystrophic lesions: histologic variability may be linked to the different stage of the disorder or reflect the severity of the disease or both [2]. The prevalence of CMDs is poorly known, and it is roughly maintained in the range of 1/100.000 individuals. In northeast Italy, as reported by Mostacciuolo [3], the prevalence has been calculated to be around 0.68x10^5^. Norwood et al. [4] in a study carried out with the aim to assess the prevalence of the genetic muscle disease in Northern England, report CMDs to be 0.76 × 10^5^. Recently, Graziano et al [5] in an Italian study population report a total CMDs prevalence of 0.563 × 10^5^, specifically, in a total of 336 patients enrolled in the study, 135 (40.2 %) showed alpha-dystroglican glycosylation deficiency with a point prevalence 0.226 × 10^5^; laminin alpha 2 deficiency was reported in 24.1 % with point prevalence of 0.136 × 10^5^; collagen VI deficiency was registered in 20.24 % with a point prevalence of 0.114 × 10^5^. Mutations in *SEPN1* and *LMNA* were less frequently reported: 6.25 and 5.96 %, with point prevalence of 0.035 × 10^5^ and 0034 × 10^5^, respectively.

### Past and more recent CMDs classification

Several classifications have been proposed to distinguish the different forms of CMDs. Parano et al. [6, 7] distinguished different CMDs: 1) Classical CMD (without mental retardation) a) with merosin deficiency and b) with normal merosin; 2) CMD with forebrain malformations-mental retardation: a) Cobblestone lissencephaly syndromes including Fukuyama congenital muscular dystrophy, Muscle-eye-brain disease, Walker-Warburg syndrome, and isolated Cobblestone lissencephaly; b) CMD with occipital agyria; 3) CMD with mental retardation (not otherwise classified); 4) Atypical CMDs: a) Autosomal Dominant CMD, b) CMD with central nervous system malformations and normal intelligence including patients with cerebellar hypoplasia/atrophy and patients with syringomyelia; c) CMD with prominent extra-CNS manifestations with Hirschprung disease and patients with severe cardiac disease; d) other atypical syndromes including Ullrich syndrome/rigid spine/”atonic sclerotic” CMD.

A classification of CMDs based on the combination of clinical, biochemical, molecular, and genetic findings was proposed by Voit and Tomè in 2004 [8] who distinguished CMDs in four groups: 1) deficit of laminin alpha 2 (formerly known as “merosin”), primarily affecting the basement membrane (MDC1A); 2) defects caused by an abnormal glycosylation of alpha-Dystroglycan: Walker Warburg syndrome (WWS), Muscle-Eye-Brain diseases (MEB), Fukuyama CMD (FCMD), and MDC1B, MDC1C, MDC1D; 3) disorders producing prominent contractures, rigid spine, and Ullrich CMD; 4) primary or secondary alpha 7 integrin deficiency.

More recently, Bonneman et al. [2] reported on the currently recognized of CMD entities: a) laminin alpha 2 related CMD (primary laminin 2/merosin deficiency); b) alpha-dystroglycan related dystrophies; c) congenital disorders of glycosylation (CDG) with abnormal alpha-dystroglycan glycosylation; d) collagen VI and Integrin-related CMD forms; e) Intracellular and nuclear CMD forms. In the group of alpha-dystroglycan related dystrophies, the authors include a) CMD with partial merosin deficiency (MDC1B with locus in 1q42); b) LARGE related CMD (MDC1D—gene *LARGE*); c) Fukuyama CMD, *FCMD* gene, protein Fukutin; d) Muscle-Eye-Brain (MEB) genes *POMGnTI, FKRP, ISPD, TMEM5,* Fukutin); e) Walker-Warburg syndrome (WWS), genes *POMTI, POMT2; FKRP, ISPD, CTDC2, TMEM5, POMGnTI, B3GALNT2, GMPPB, B3GNT1, SGK196,* Fukutin); e) CMD/LGMD with Mental Retardation (genes *FKRP, POMTI, POMT2, ISPD, GMPPB*); f) CMD/LGMD without mental retardation including MDC1C (genes *FKRP, ISPD, GMPPB,* Fukutin).

Kang et al. [9], following the establishment of the Guideline Development Subcommittee of the American Academy of Neurology and the Practice Issues, distinguished the types of CMDs in the following major categories: collagenopathies autosomal recessive (AR) and autosomal dominant (AD) (also known as Collagen VI related myopathies), including Ullrich CMD and Bethlem myopathy; merosinopathies AR (also reported as merosin-deficient CMD); dystroglycanopathies AR (alpha-dystroglycan-related MDs) including FCMD, MEB, WWS, and primary alfa-dystroglycanopathy, MDDGA10, MDDGA11, MDDGA14. In the group of unclassified CMDs, the authors include rigid spine syndrome (*SEPN1* and *FHL1* genes), multiminicore disease (*SEPN1*), and LGMD (*LMNA* gene).

Classifications, clinical features, and genotype/phenotype correlation in CMDs are constantly in progress due to the worldwide use of molecular analysis and an usually prolonged survival of the affected patients due to a more suitable clinical assistance.

In this article, we want to point out our attention to CMDs belonging to the group of dystroglycanopathies (also reported as alpha-dystroglycan related MDs) [2] and in particular on clinical features of the most severe disorders of this group: FCMD, MEB disease, and WWS. An infant with severe congenital hypotonia represents a great clinical challenge for pediatricians. Establishing the events that have caused the lesions, the difficulties in localizing the involved CNS (central vs peripheral), which in some circumstance may concur, difficulty in performing and interpreting diagnostic procedures, particularly in the early age, and distinguishing the different and numerous types of peripheral neonatal hypotonia including the different group of CMDs are all difficult tasks for pediatricians who treat these children. An approach to the clinical diagnosis to the different form of CMDs and related conditions is reported.

### defective glycosylation of alpha-dystroglycans

Defective glycosylation of alpha-dystroglycans is one of the events that may cause CMDs. These disorders encompass several conditions that range from early and severe clinical involvements, as demonstrated by the WWS, MEB, and FCMD, to later and less pronounced muscle impairment and with/without comorbidity with other body-organs [10–12]. Clinical presentation of this group of disorders is heterogeneous, and this has led researchers to consider them as a wide spectrum of the same entity.

Several genes have been implicated in the pathogenesis of the alpha-dystroglycanopathies. Among these, *POMT1, POMT2, POMGnT, FCMD, Fukutin,* and *LARGE* genes are reported to code for specific or putative glycosyl-transferases involved in the pathway of alpha-dystroglycanopathies.

A multicenter Italian study carried out by Mercuri et al. [13] in 81 CMDs with defective glycosylation of dystroglycan gave the following results: WWS was diagnosed in six with mutations identified in 4 (*POMT1* in two, *POMGnT1* in one, *LARGE* in one); MEB/FCMD-like in 24 with mutations identified in 13 (*POMGnT1* in six, *FKRP* in three, *POMT1* and POMT2 in two, respectively); CMD with cerebellar involvement in 15 with mutations identified in 8 (*POMT1* in four, *POMT2* in three, *POMGnT1* in one); CMD with mental retardation and normal MRI in 21 with mutations identified in 10 (*POMT1* in five, *POMT2* in three and *FKRP* in two); CMD with no mental retardation and normal MRI was reported in 4 patients with mutations identified in 3 (*POMT*, *FKRP* and *Fukutin,* in one patient each).

CMDs are frequently associated to intellective delay (ID) and structural brain involvement and Messina et al [14] report a figure of 92 out of 160 CMDs patients with cognitive impairment: patients affected by alpha-dystroglycan deficiency were the majority (73/92, 79 %), mostly harboring mutations in *POMGnT1* gene (42/73, 57.5 %).

### Fukuyama Congenital Muscular Dystrophy (FCMD)

FCMD was first reported by Fukuyama [15] in 1960. The gene responsible for FCMD was found to be located on chromosome 9q31-33 (*FCMD* or *FKTN*) [16]. Its product, named fukutin, was identified by Kobayashi et al. 2 years later [17]. Fukutin is a putative glycosyltransferase of 461 aminoacids. The clinical features of FCMD reported in 15 patients by Fukuyama et al. [15, 18] were severe and consisted of hypotonia with an extremely early onset and a generalized distribution of the affected regions, including facial muscles and proximal muscles slightly more severely affected than the distal. Joint contractures at an early stage such as those found in congenital multiple arthrogryposis, and hypokinesia were also reported. Moreover, some patients presented with mental and speech retardation, febrile or afebrile seizures, and pseudohypertrophy of the skeletal muscles (found in about 50 % of the patients). Severe functional disabilities and very slow progressive course were also found. At the muscle biopsy, the patients presented with severe degeneration and atrophy of the muscular fibers and a high degree of proliferation of the connective tissue: in particular, the proliferation was located in the endomysium, differently from what usually happens in DMD, in which an initial proliferation of the perimysium is found [18]. Pre-requisite diagnostic criteria established by Fukuyama et al. [15, 18] included the following: both sexes equally affected, onset before the age of 8 months; delayed motor development, generalized symmetric weakness and hypotonia, decreased or absent deep tendon reflexes; facial muscle involvement; elevated serum CK activity; and muscle involvement with histologic features compatible with muscular dystrophy. The head circumference was reported by these authors as small, and none of the patients showed a head circumference above the average. Cerebral involvement was also impressive with mental retardation and speech delay manifesting in more than half of the patients. The CT scan, performed in 22 patients by Fukuyama et al. [18], showed a decreased radiodensity of white matter (marked in 18 % and mild in 23 %). Other anomalies within the CT scans consisted of dilatation of lateral (59 %), third (41 %), and fourth ventricles (28 %), cortical sulci (50 %), longitudinal cerebral fissure (45 %), Sylvian fissure (91 %), and posterior cerebellar incisure (25 %). Kamoshita [19], using brain MRIs in FCMD patients, reported anomalies consisting of micropolygyria of the cerebrum and cerebellum with loss of cytoarchitecture, aberrant, and deviated pathway of the pyramidal tract. No patients with hydrocephalus were reported. From the ocular investigation in 20 FCMD patients, Honda et al. [20] reported frequent myopia and weakness of the ocular and orbicular muscles and in some cases optic nerve atrophy.

During the following years, the clinical features of the FCMD, as initially reported by Fukuyama et al., substantially have not changed. It appears evident that the affected patients show a variable degree of clinical manifestations, including variability into the members of the same family. The muscle involvement appears most frequent before and during the neonatal age with weakness and hypotonia and the history of poor fetal movements. The newborns appear floppy, and the cry is feeble. Usually, the look is vivid. Pulmonary infections may be frequent in the first months of life. Hypertrophy of the calves and quadriceps, cognitive delay, seizures, and difficulty in walking may later be present. Ophthalmologic examination may show retinal folding, fusion, focal dysplasia, and detachment [21]. The brain MRI may present with transient white matter abnormalities, occipital cobblestone cortex, hypoplasia of the pons and cerebellar vermis [22], and cerebral and cerebellar micropolygiria, as previously reported by Fukuyama et al. [18]. The main clinical features of FCMD have been recently summarized by Bonnemann et al. [2] and consist of high prevalence in the Japanese population, inability to walk, epilepsy and mental retardation, clinical overlap with MEB syndrome, but less severe eye involvement. Lissencephaly type II/pachygyria, hypoplastic brainstem, and cerebellar abnormalities are the most frequent CNS imaging findings [2]

### Muscle eye brain (MEB) disease

Muscle-eye-brain is known as “MEB disease” and is characterized by CMD, structural eye abnormalities, and cerebral malformations, first reported by Santavuori et al. in 1978 [23]. The authors reported on a series of 9 children (8 males, 1 female) and two adult siblings. The patients came from two different areas of Finland, and for some years the disorder was maintained to affect only the Finnish population. In their first presentation, the authors referred that patients with this disorder had poor psychomotor development, hypotonia, and ocular symptoms, including nystagmus with suspicion of glaucoma. The age of referral was reported as an average of 2.2 months. Tendon reflexes were normal at birth but weak or absent by 1 year of age. The motor development was drastically retarded. Only one of the children and the two adult patients could sit or move around; in one of the reported children, the acquired condition was lost after a prolonged convulsive episode. All patients suffered from convulsions, and two presented with myoclonic jerks. The head circumference developed normally with only one exception. In some of the patients, facial dysmorphism was also reported: the forehead was high and prominent with a wide fontanel, the mid-face was flat, and the nose and filtrum short. The patients suffered from severe visual failure with congenital glaucoma and hypoplastic choroid; optic atrophy was found in 7 patients and optic coloboma in 3. The laboratory investigations displayed an increased level of serum creatine phosphokinase (CK) present since the neonatal period and persisting elevated in all children with values ranging from 150 to 1550 IU/ml. The EMG showed slight myopathic changes with progressive course during the ages. Muscle histology was similar in all patients and consisted of typical signs of muscular dystrophy at various stages. A few years after the Santavouri’s report [23], Dambska [24] described the “cerebro-ocular muscular syndrome” on three siblings who manifested with dysmorphic face, hypotonia, areflexia, and failure to thrive. In these patients, eye anomalies consisted of corneal opacity, cataracts, and dysgenesis of the anterior chamber. Agyric hemispheres, polymicrogyria in several cortical segments, and severe cortical disorganization were the cerebral malformations reported at MRI. The muscle biopsy disclosed a severe muscular dystrophy. The authors [24] defined these patients as affected by a variant of Fukuyama congenital cerebro-muscular dystrophy, but the clinical features of the patients recall those of MEB disease. Patients with similar clinical features of MEB were also reported by Korinthenberg et al. [25] in three German patients, demonstrating the wide diffusion of this condition that affected not only patients of Finnish origin.

The clinical and molecular aspects of MEB disease are typical and specific, but the grade of severity of each organ affected is quite variable. The disorder is inherited in autosomal recessive pattern and is associated with mutations in the gene at 1p34-p32 that codifies POMGnT1, a glycosyl transferase. All the mutations in *POMGnT1* cause a complete loss of function of the glycosyltransferase, but, as reported by Hehr et al. [26], the type of mutation appears to not be related to the variability in clinical severity. As reported in the literature, the most frequent clinical anomalies reported in MEB are the congenital muscular dystrophy, constantly observed and sometimes as a single (pure) manifestation: the eye anomalies are usually congenital and may include severe myopia, glaucoma, optic nerve, and retinal hypoplasia. The cortical malformations may be present with defective migration ranging from pachy-polymicrogyria and agyria, white matter abnormalities, and anomalies of the brainstem and cerebellum [26].

Taniguchi et al. [27] reported on patients first diagnosed as FCMD, MEB, or WWS, respectively. They demonstrated that MEB is caused by loss of function mutations in the gene encoding protein O- linked mannose Beta 1 2-N-acetylglucosaminyltransferase I (POMGnT1). The disorder is present in different races with a worldwide distribution, and it has a broader clinical spectrum than previously expected. In a recent survey in Turkish patients, Yis et al. [28] from a cohort of 34 patients affected by CMD, reported twelve patients who presented signs diagnosed as MEB disease. The most common mutation reported in 66 % of patients was c.1814G > A (p. R605H). Two novel mutations were identified by these authors, who referred that all the patients showed muscular hypotonia at birth followed by variable degree of spasticity and exaggerated deep tendon reflexes, secondary to brain involvement. In the later stage of life, the mean serum CK values were 2485.80 + - 1304.54 IU/L (700 to 4267 IU/L). The most common ophthalmologic and cerebral anomalies consisted of cataracts, retinal detachment, periventricular white matter abnormalities, ventriculomegaly, pontocerebellar hypoplasia, and multiple cerebellar cysts.

Falsaperla et al. reported a 13 year-old girl with a long-term survival, demonstrating the wide variability of MEB disease [29]. The molecular analysis displayed mutations in the *PDHGT1* gene. The child showed failure to thrive with height and weight under the 3^rd^ percentile and a head circumference around the 3^rd^ percentile. Her neurological examination showed speech delay, strabismus, proximal and distal weakness with hypotonia of the upper limbs, and signs of hypertonia in the lower limbs with slight contracture of the fingers and wrist. The eyes presented with severe myopia and retinal dysplasia. The MRI showed cortical gyration of the frontal lobes, micropolygyria of the temporal lobe, and bilateral microcystic degeneration of the cerebellar cortex. The course presented by this girl was milder than usually reported and is a confirmation of the variable clinical aspect of the disorder. As reported by Bonnemann [2], MEB disease may involve different genes such as *POMGnTI, FKRP, Fukutin, ISPD, TMEM5* with clinical features in summary consisting of mainly severe congenital weakness, inability to walk, spasticity and motor deterioration, mental retardation, and severe eye involvement. The brain MRI reflects what has been reported in FCMD with lissencephaly type II/pachygyria, brain stem and cerebellar anomalies and cysts.

### Walker Warburg Syndrome (WWS)

The syndrome is named after Walker [30], who described a patient with lissencephaly, and Warburg [31] who reported, in 1978, a 6-year old boy who was severely retarded, hypotonic, and unable to sit up even with support. As described by Warburg [31], the boy was born from consanguineous parents (first cousins). During the infancy, the child suffered from unexplained diarrhea and failure to thrive. His right eye was microphthalmic with retinal detachment and hydrocephalus was also present and treated with a shunt. In the first year of life, he developed scoliosis and secondary contractures of arms and legs. At the time of the admission, Warburg reported that the child was blind and severely mentally retarded with a communicating hydrocephalus, right microphthalmia with congenital retinal detachment, and a left abortive falciform fold.

A family was reported from Catania, Italy, in whom three members suffered from congenital hydrocephalus and ocular abnormalities [32]. In two of them, the muscular tone was normal, while the third presented with cerebral, eye, and muscle involvements. The patient was born by caesarean delivery with hydramnios. The mother mentioned that fetal movements were present but feeble. The head circumference increased rapidly since the first days of life and reached the diameter of 42 cm at 1 month. Severe muscular hypotonia was present with the first three fingers flexed and absence of deep tendon reflexes and muscle enzymes were increased. At autopsy, the child showed a classic lissencephaly with midline fusion of frontal lobes with absence of lobi olfactorii. Coronal sections showed widespread cortical malformations consisting of agyria, pachygyria, and/or micropolygyria with many subcortical ganglion heterotopias. The histological evaluation of muscle affected showed a severe degeneration of muscle fibers with severe interfascicular fibrosis and lipomatosis (Fig. 1). The authors concluded that the reported patients could represent a clinical variable of the Warburg syndrome and suggest some links with Fukuyama congenital muscular dystrophy and/or with the so-called brain-eye-muscle disease of Santavuori. The same authors [32] were among the first to anticipate and to suggest that the three syndromes (Walker-Warburg, Fukuyama, and “Santavuori”) could be considered as three variants of the same disease.

**Fig. 2:**
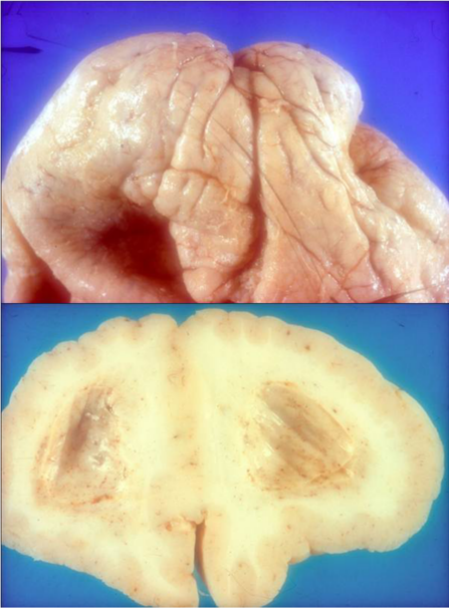
Archive’s photo. The same patient. Macroscopic examination, showing severe architectural disarrangement with clear signs of lissencephaly

**Fig. 3:**
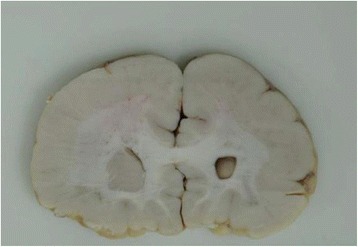
A new case of WWS showing severe lissencephaly lesions

WWS is reported to be genetically heterogeneous. The genes involved are *POMT1*, *POMT2*, and less frequently *POMGnT1*, *FKRP*, *Fukutin,* and *LARGE* [33–37]. The clinical picture of this disorder is particularly severe as most of the children do not survive beyond the first years of life. A clinical description of the disorders has been reported by Dobyns et al. [34] with detailed clinical description of the disorder; aside the severe congenital muscular dystrophy, the brain malformation is constantly present and represented by lissencephaly type I with cobblestone cortex, obstructive hydrocephalus, neuronal heterotopias, corpus callosum agenesis, fusion of the hemispheres, and ponto-cerebellar hypoplasia with fourth ventricle dilatation (Figs. 2 and 3). Occipital encephalocele and Dandy Walker cyst may be present. The eye anomalies involve both anterior and posterior chambers with retinal detachment and blindness. Microphthalmia, thalamus, optic nerves hypoplasia, colobomas and iris malformation, cataract, and megalocornea may be also found. The clinical features may be also associated, even if not frequently, with facial dysmorphism and cleft lip or palate [38].

### Other less known types of CMDs belonging to alpha-dystroglycan related dystrophies

#### Congenital muscular dystrophy type 1C (MDC1C)

This subtype of CMD was first reported by Brockington et al. [39], who identified a new member of the fukutin family of proteins, FKRP, with the gene mapping to chromosome 19q13.3. The disorder manifests in the first few weeks of life with CMD and marked increase of serum CK. Several patients present with normal intelligence and normal brain structures on brain imaging. In the young adult age, the patients show heart involvement, severe weakness, and wasting of the shoulder girdle muscles; hypertrophy and weakness of the leg muscles with calf and thigh hypertrophy and severe respiratory failure are also recorded. The MDC1C follows the nomenclature previously established for the MDC1A related to CMD with a primary laminin apha 2 deficiency and for MDC1B related to a secondary laminin alpha2 deficiency and calf hypertrophy linked to 1q42. The group MDC1C also includes clinical features of CMD/LGMD involving different genes (*FKRP, Fukutin, ISPD, GMPPB*), which manifests with early onset weakness and early onset LGMD without brain involvement and cardiomyopathy.

#### CMD with partial merosin deficiency (MCD1B)

This form manifests with variable deficiency of the glycosylated aDG epitope and secondary laminin alpha 2 deficiency. The muscle involvement manifests with proximal limb girdle weakness, muscle hypertrophy, particularly in the calf, and early respiratory failure [40].

#### *LARGE* related CMD (MDC1D)

This disorder has been initially reported by Longman et al. [41] in a 17-year-old girl who displayed weakness some months after birth and manifested severe mental retardation. Failure of brain migration and white matter anomalies with abnormal electroretinogram were also reported. This anomaly may disclose some clinical features of MEB and/or WWS.

The muscle biopsy in the case of Longman et al. [41] showed a severe dystrophic pattern and a decreased alpha-dystrogycan at immunolabeling.

A few years ago, two Sicilian siblings, a boy and a girl, born from consanguineous parents with congenital muscular dystrophy and peculiar phenotype were reported by Voit et al. [42]. In these patients, the genetic causes were unknown, but several investigations with linkage analysis allowed the authors to exclude the candidate loci on chromosomes 1q32, 6q2, and 9q31. The gene locus for MCD1B on chromosome 1q42 was also excluded as was laminin alpha 2, laminin beta 2, and alpha dystroglycan, which showed normal results at immunocytochemistry of the muscle membrane proteins. The siblings showed severe generalized muscle weakness, including facial muscles, marked level of CK (ranging from 200 to 700 U/L), and histological alterations compatible with muscular dystrophy. In addition, both children showed a peculiar phenotype with adducted thumbs and toe contractions. Ptosis, external opthalmoplegia, mild mental retardation, and mild cerebellar hypoplasia on MRI were reported [42].

#### Neonatal hypotonia: a challenge to reach the diagnosis

As previously mentioned, the causes of the neonatal hypotonia are several and to recognize the events that have provoked the disorders represents a great challenge for the pediatricians. Several conditions may manifest with infantile hypotonia, including anomalies of the central or peripheral nervous system with involvement of the spinal cord, anterior horn cell, peripheral nerves, neuromuscular junction, and muscles. The term “floppy Infant” is referred to as the most severe clinical form of hypotonia, and it is related to a child of an age ranging from first days to 1 year of life, presenting with severely reduced or absent muscle mobility.

#### History and clinical examination

**Fig. 4:**
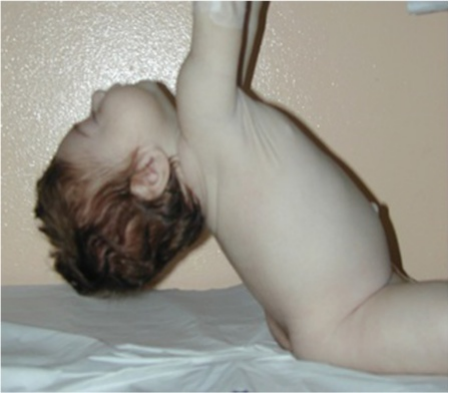
Physical examination of the hypotonic child: “Pull to sit” maneuver

**Fig. 5:**
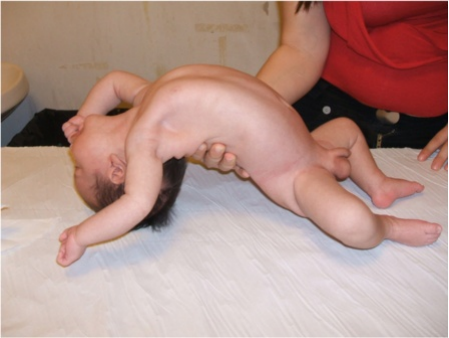
Physical examination of the hypotonic child: “horizontal suspension” maneuver

**Fig. 6:**
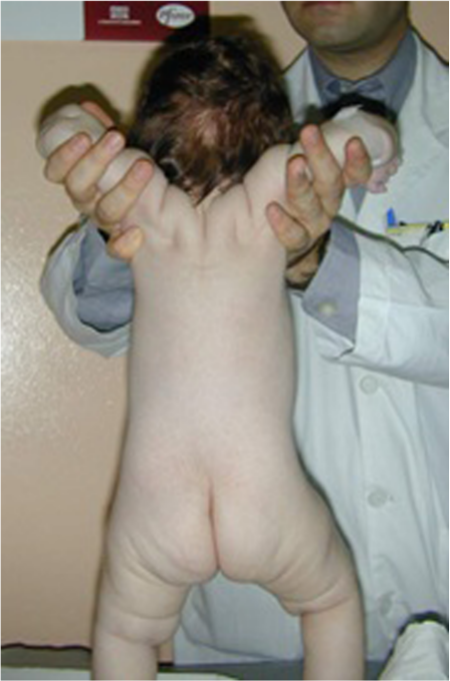
Physical examination of the hypotonic child: “vertical suspension” maneuver

The diagnostic evaluation includes detailed family, pregnancy and, perinatal history. General physical examination to exclude malformative anomalies and body structural defects. The muscle tone is evaluated according to the standard maneuvers for hypotonic infant such as “pull to sit”, the “horizontal” and “vertical suspension” (Figs. 4, 5 and 6). Suggestive of a prenatal origin of the hypotonia in infancy are the following: family history (consanguineity, familial muscle disorder); pregnancy history (reduced fetal movements, polyhydramnios, vertex presentation); general physical examination at birth (joint contractures, pes cavus, skin dimples, sucking and swallowing difficulty, reduced or absent patellar reflexes). Physical examination is also directed to exclude malformative anomalies involving face, extremities, heart and genital organs and classical syndromic disorders [43].

The distinction between central and peripheral nervous system attainment may be only indicative since both these structures may be clinically affected, and both structural anomalies may co-exist in the same affected infants in a variable expression. In general, severe weakness with reduced or absent antigravitary movements, poor spontaneous movements, absent tendon reflexes, marked head lag and alert look are consistent with hypotonia of peripheral origin. Evaluation of the cry, suck, facial expression, antigravity movements (as clinical clue of muscular strength), resistance to strength testing, and respiratory capacity are also to be established [44]. Infants with central involvement tend to show an unexpressive look, usually normal strength with hypotonia and increased or normal patellar reflexes, facial dysmorphism, malformative anomalies neonatal seizures, and history of neonatal distress. However, the clinical examination in the case of a floppy infant needs to be supported by laboratory investigations including assessment for intrauterine or perinatal infections, TORCH, hemogram, blood gas analysis, erythrocytes sedimentation rate, and PCR. Investigations for muscle disorders include creatine kinase (CK), aldolase, alanine aminotransferase (ALT), or aspartate aminotransferase (AST). Other recommended investigations include ammonia, lactate, aminoacids, organic acids, very long chain fatty acids, carnitine/acylcarnitine profile, 7 dehydrocholesterol, isoelectric focusing of the sialotransferine, N- and O- glycan analysis, and galactose 1- phosphatase that may lead to the diagnosis of hypotonia of metabolic origin. An increased CK, aldolase, ALT and AST are usually indicative of a dystrophic process. The diagnosis of Prader-Willi syndrome should be considered and excluded through the methylation analysis of the chromosome region of 15q11-q13. Specific lysosomal enzyme testing for Pompe disease should be performed when hypotonia is associated to cardiomyopathy. EMG and NCV in infancy are technically difficult to be carried out, and the interpretation of the data remain sometime doubtful. These investigations are not as conclusive for the diagnosis as they may be at older age. EMG could be useful in distinguishing central vs. peripheral neuropathy or muscle involvement. A neurogenic pattern shows increased amplitude of action potentials; a myopathic pattern is characterized by reduced action potentials and increased interference; a myotonic pattern shows increased insertional activity, while a myasthenic pattern an abnormal repetivity. EMG is not advisable in case of suspected spinal muscular atrophy (SMA): clinical examinations, decreased deep tendon reflexes and the absence of antigravitary movements are classically suggestive for the diagnosis. NCV may be useful in displaying hereditary motor sensory neuropathies. Muscle biopsy is advisable since the basic histology may show signs of myopathic, neuropathic, or dystrophic changes. Use of Immunohistochemical techniques and electron microscopy may be worthy to identify the types of congenital myopathies and to distinguish different types of CMDs.

Ophthalmologic evaluation, electrocardiogram, electroencephalogram, and brain MRI should also be carried out according to the clinical examination.

Among the group of floppy infants of peripheral origin, differential diagnosis is also difficult, including non-neuromuscular genetic conditions such as Prader-Willi syndrome [45]. Hypotonia and weakness are characteristic signs present aside CMDs, in very early SMA, in congenital myotonic dystrophy, in congenital myasthenic syndromes, and in congenital myopathies. These disorders need to be distinguished from classical CMDs (FCMD, MEB, WWS), and the last one must be differentiated by the different types and subtypes of CMDs [45].

**Fig. 7:**
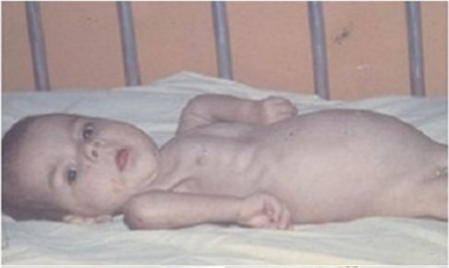
Infant with early SMA. Note the severe hypotonia, the vivid look, severe involvement of diaphragm, and intercostal muscles

Very early Spinal Muscular Atrophy (Fig. 7) is inherited with autosomal recessive modality. The neonatal patients show hypotonia and weakness mainly proximal and symmetrical with fasciculations of the tongue and intention tremor. The look is vivid, and the deep tendon reflexes are absent. Diagnosis is linked to SMN1 copy number variation.

**Fig. 8:**
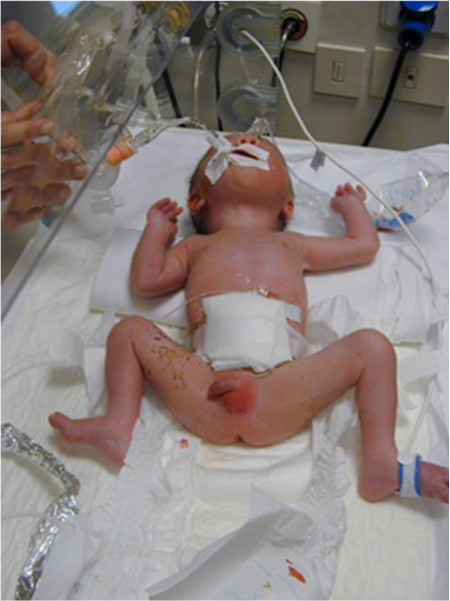
Severe myotonic dystrophy. Patient born with polyhydramnios with congenital talipes equino-varus responsive to Mestinol

Congenital Myotonic Dystrophy (Steinert disease) (Fig. 8) is the most frequent neuromuscular disorder in the neonatal period. It is caused by a CTG-repeat expansion in DMPK. The pregnancy history displays reduced fetal movements and poli-hydramnios. Clinical signs are present at birth. Affected infants show deficit of diaphragm and intercostal muscle functions and facial diplegia. Arthrogryposis and club feet may be noticed since birth. The EMG is usually not diagnostic at birth, but it may give a positive result subsequently. Multiple RNA splicing defects have been recognized, leading to classify this disorder as a “spliceopathy” [46, 47].

For the congenital myasthenic syndrome, two forms are classically recognized: 1) Myasthenia Gravis is the transient, acquired neonatal myasthenia present at birth or within the first 3 days of life. The hypotonia is generalized and severe. The patients show feeding difficulties with failure in sucking and swallowing, facial weakness, and feeble cry. Ptosis and ophthalmoplegia are frequently present, and these signs may be useful for obtaining a correct diagnosis.

2) The Congenital myasthenia manifests with failure of the bulbar, ocular, limbs, and respiratory muscles. ECG may show heart block. EMG may be useful for diagnosis. The response to treatment with acetylcholinesterase inhibitors is diagnostic.

**Fig. 9:**
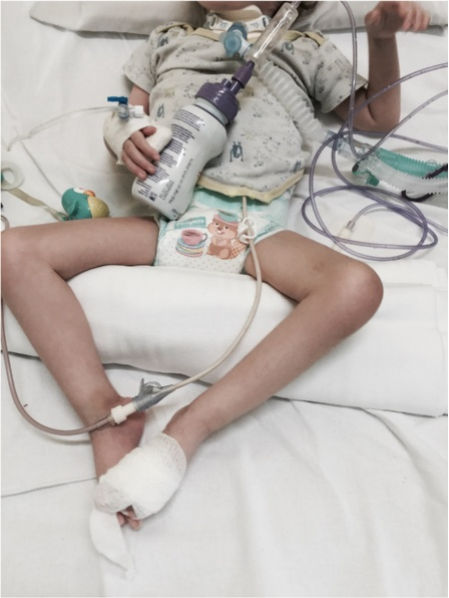
A girl, 2 years old, affected by nemalinic myopathy

The patients with congenital myopathies (CMs) (Fig. 9) present at birth with severe generalized muscle weakness, facial weakness, and ophthalmoparesis. Peculiar structural or developmental abnormalities in absence of dystrophic lesions in the muscles are the histopatological features. The most known forms include the following: centronuclear myopathy-including myotubular myopathy) (*MTM1, DNM2, MYF6, CCDC78, BIN1* genes); core myopathy including minicore (*RYR1*, SEPN1, TTN, MYH7 genes); nemaline myopathy (TPM3,NEB,ACTA1, TPM2, TNNT1, KBTBD13, CFL2 genes); congenital fiber-type disproportion ACTA1, SEPN1, TPM3, TPM2, MYL2 genes). In these disorders, the histochemistry or electron microscopic investigations are diagnostic [46].

The muscle biopsy, together with specific molecular/genetic examinations, remains the gold standard for the diagnosis of specific CMDs and similar conditions. Muscle analysis is worthy for obtaining a correct diagnosis. The analysis should include the general histologic structure, histochemical and immunohistochemical reactions, and possibly specific enzyme quantification. In some cases, the investigation should be extended to electron microscopy analysis.

#### Treatment

At the present time, the treatment is led to ameliorate the course of the disease and to prevent or treat pulmonary and cardiac impairment. Antisense oligonucleotide has been shown to have a good response in the mouse model for myotonic dystrophy, and it is in course of trial in humans [48]

There is no pharmacological treatment for the CMDs. However, physiotherapic treatment is advisable to prevent joints deformity, muscles retractions, and scoliosis. Supportive treatment with non invasive respiratory support in case of respiratory distress, correction of gastro-esophageal reflux, support in cardiac failure, treatment of respiratory infections, and nutritional treatment with new modality must be constantly carried out. This treatment strategy has allowed these patients to a wider and better survival.

## Conclusions

CMDs are a group of disorders that involve more than only muscles; other body structures including the brain, eyes, and heart may be affected. Diagnosis is not easy, and the time when a muscular biopsy should be performed is not easy to be established. However, muscular biopsy becomes worthy to indicate the correct molecular/genetic analysis. The course of any of these forms is usually severe. Nutritional and respiratory support have prolonged the life of these children, but early fatal outcome is typical. Performing clinical and molecular diagnosis is extremely important for genetic counseling, prognosis, and anticipatory treatment and also for prospective treatment.

## Abbreviations

CMD: Congenital muscular dystrophy
DMD: Duchenne muscular dystrophy
FCMD: Fukuyama congenital muscular dystrophy
LGMD: Limb-girdle muscular dystrophy
MD: Muscular dystrophy
MEB: Muscle-eye-brain diseases
WWS: Walker-Warburg syndrome
